# Commentary on: Infraorbital Hyaluronic Acid Filler: Common Aesthetic Side Effects With Treatment and Prevention Options

**DOI:** 10.1093/asjof/ojac009

**Published:** 2022-02-01

**Authors:** Mark G Albert

**Affiliations:** Manhattan Eye, Ear and Throat Hospital of Northwell Health, New York, NY, USA

This was a study involving 800+ patients over 8 years in the author’s practice. Infraorbital filler is a challenging procedure often avoided by many well-trained physicians due to high complication rates.^1^ This article goes into detail about optimal technique as well as prevention and management of the most common complications. There are several important points from the article worth reiterating (Video).

The author indicates that the best candidates are those that require <0.5 cc total per side. It is prudent practice to follow this as a guideline to minimize complications. If needed, patients can return in 1 month for additional filler beyond this volume. The author also recommends only using fillers with low hydrophobicity like Restylane (Galderma, Lausanne, Switzerland), Volbella (Allergan, Dublin, Ireland), or Belotero (Merz, Frankfurt, Germany). Using a filler with higher hydrophilicity is an invitation for increased edema and thus a blue hue in the undereye area.

The article mentions avoiding entry points in the malar eminence as this can cause malar mounds. This is certainly an accurate observation, and therefore an upper nasolabial fold entry point, with or without an additional lateral entry site, is optimal to treat the entire undereye area.

The blue hue that can be seen after filler is actually not from the Tyndall effect but instead from the Rayleigh effect.^1^ Tyndall proponents attribute the scattering of blue light from superficial filler particles. The issue is that the filler particles should be approximately the same size as the light for it to happen (400-700nm; smaller than 1 micron) but filler particles are 250-1000 microns in size. The Rayleigh effect attributes the blue hue to swelling because the particles in edema are in fact less than 1 micron. So, it is important to understand this and therefore try treatment with triamcinolone before hyaluronidase.

The author accurately mentions that post-procedure “lumps” are usually not from the filler itself but instead from veins that become distended after treatment. It is important to prepare patients ahead of time and explain that periorbital veins are best treated with a laser^2^ (such as Nd:yag) or even a periorbital phlebectomy.^3^ Sclerotherapy in this area has caused retinal artery occlusion in some reports.

As far as the shortcomings of the article, the author states that patients report less pain, edema, and bruising with a cannula compared with a needle. Data on such a claim would be beneficial because cannulas can be quite pain and anxiety provoking in the periorbital region specifically, especially when piercing the robust orbital retaining ligament.

Additionally, regarding the author’s statements about undereye ecchymosis, the degree of bruising is technique dependent. Injecting with a needle directly through the lower lid skin produces the most ecchymosis, whereas a nasolabial, lateral, submalar, or even transoral approach avoids venous injury and thus provides for a better post-procedure experience for the patient.

This is a valuable contribution to the literature that presents many teaching points even for experienced injectors. I applaud the author for sharing her extensive experience with the readers of *ASJ Open Forum*.
